# Lack of mutagenicity, genotoxicity and developmental toxicity in safety assessment tests of *Lactobacillus mali* APS1

**DOI:** 10.1371/journal.pone.0208881

**Published:** 2018-12-13

**Authors:** Yu-Chun Lin, Yung-Tsung Chen, Ming-Ju Chen

**Affiliations:** 1 Livestock Research Institute, Council of Agriculture, Executive Yuan, Tainan, Taiwan; 2 Department of Animal Science and Technology, National Taiwan University, Taipei, Taiwan; 3 Institute of Biotechnology, National Taiwan University, Taipei, Taiwan; Universita degli Studi di Napoli Federico II, ITALY

## Abstract

*Lactobacillus* (*L*.) *mali* APS1 isolated from sugary kefir grains has been proven to affect energy and glucose homeostasis. However, without proper safety assessment it cannot be recommended as probiotics for human consumption. For genotoxicity, the Ames test showed no mutagenic effect of *L*. *mali* APS1 in the presence or absence of S9 mix metabolic activation. *In-vitro* mammalian chromosomal aberration test showed that the number of Chinese hamster ovary cells with abnormal chromosomes was <5% after *L*. *mali* APS1 treatment. Moreover, *L*. *mali* APS1 showed no risk of genotoxicity potential compared to the control. *L*. *mali* APS1 administration did not cause significant (p>0.05) changes in body weight, the number of reticulocytes, or in the occurrence percentage of micronucleus in Imprinting Control Region (ICR) mice. Based on the absence of maternal or fetal effects at any dosage level investigated, the teratogenicity could be defined as greater than 1,670 mg/kg b.w./day for maternal general toxicity and fetal development when *L*. *mali* APS1 was orally administered by gavage to pregnant SD rats during gestation days 6 to 15.

## Introduction

Lactic acid bacteria (LAB) are a functional group of microorganisms with a long history of safe use in fermented food production and animal feed [1, 2]. Among known LAB, the *Lactobacillus* genus includes a high number of GRAS (Generally Recognized As Safe) species, conferring health benefits on the host as probiotics [3]. In addition, 36 *Lactobacillus* strains received qualified presumption of safety (QPS) status in the European Union [4].

Sugary kefir is a homemade health-promoting beverage commonly manufactured with sugary kefir grains [5–7]. These grains are small, transparent mucilaginous masses that consist of a polysaccharide gel that contains embedded LAB and yeasts, which can be cultivated in a solution of sugar and water [7, 8]. The beneficial effects of sugary kefir strains on human health have been proven in the context of symbiotic mixture, but have not yet been scientifically substantiated individually [9].

A novel LAB strain isolated from sugary kefir grains, *L*. *mali* APS1 is a Gram-positive (0.5–1 μm in length), non-spore forming, facultative heterofermentative bacterium [7]. Few studies have focused on the biological or functional properties of *L*. *mali* APS1. Nevertheless, to date there remains a void in the literature on the characterization of possible mechanisms that this strain imparts.

Our previous study suggested this bacterium as a probiotic due to its anti-obesity effect. It has been proven to regulate energy and glucose homeostasis in diet-induced obese mice [10]. The high-fat diet (HFD) mice orally administered with *L*. *mali* APS1 significantly reduced body weight gain, body fat, liver weight, and fat accumulation in the mesenteric adipose depot when compared with the control. Moreover, dietary supplementation with *L*. *mali* APS1 also effectively maintained the blood glucose level by increasing serum glucagonlike peptide-1 (GLP-1) [10]. In addition, *L*. *mali* APS1 could prevent non-alcoholic fatty liver disease (NAFLD) by ameliorating hepatic lipid accumulation and reducing oxidative stress activities in liver tissues of HFD-fed rats, indicating potentials in modulating gut microbiome and increasing antioxidant activities [11].

Although many species of the genus *Lactobacillus* have long been used as food preservatives, the safety data on newly isolated LAB strains remain limited and, without such, their safety might be questioned especially with the infective lesion incidence in one patient [12–14]. Therefore, the safety of newly isolated LAB strains needs to be validated prior to claiming their probiotic potential, since they may not share the safety status of traditional *Lactobacillus* strains [15–18].

In the present study, the safety of *L*. *mali* APS1 was assessed in accordance with the Organization for Economic Co-operation and Development (OECD) Guidelines, namely *in vitr*o evaluations as well as *in vivo* studies using mouse or rat models to depict the safety of the strain in question.

## Material and methods

### Ethics statement

All toxicity studies were conducted by Medgaea Life Sciences Ltd. (New Taipei City, Taiwan) to performed in accordance with Good Laboratory Practice (GLP) for Nonclinical Laboratory Studies of OECD (ENV/MC/CHEM(98)17, 1997), FDA(21 CFR Part 58, 2014) and Department of Health (DOH) in Taiwan. All test rodents were bred and purchased from Biolasco Taiwan Co., Ltd (I-Lan, Taiwan). Animal housing and handling including blood collection and sacrifice were performed in accordance with National Institutes of Health (NIH) Guide for the Care and Use of Laboratory Animals. All animal husbandry and experiments were conducted in accordance with the national legal requirements, and all studies were approved by the Institutional Animal Care and Use Committee of Medgaea Life Sciences Ltd (permit no. MG104018 and MG104014).

### Bacterial test substance

*L*. *mali* APS1 was isolated from sugary kefir and identified as previously described [10]. The *L*. *mali* APS1 powder (1×10^10^ CFU/g) was manufactured by Synbiotech Co., Ltd. of Kaohsiung, Taiwan. Briefly, *L*. *mali* APS1 was cultivated by batch fermentation with a sterilized medium consisted of proteins, carbohydrates, and minerals in water, then inoculated with the strain. After incubation, the broth was centrifuged at 5000 × g for 10 min at 4°C. Then, the supernatants were discarded, and the cell pellets were freeze-dried and stored at -20°C until use.

### Ames test

A bacterial reverse mutation assay was performed to evaluate the mutagenicity of *L*. *mali* APS1 with or without S9 activation, following the principles of OECD Guideline 471 (1997)[19]. The assay was conducted using *Salmonella typhimurium* histidine-auxotrophic strains TA97a, TA98, TA100, TA102, and TA1535 (Molecular Toxicology, INC, Boone, NC). The S9 post-mitochondrial supernatant of rat liver (Moltox, Shanghai, China) homogenate was used as a metabolic activation system.

The negative (0.1 mL distilled sterile water) and positive controls were showed in Table 1. Different dilutions of *L*. *mali* APS1 samples (5.0, 2.5, 1.25, 0.625, and 0.3125 mg/plate) were used for all tests under the same conditions.

**Table 1:** Positive control of Ames test.

TA strain	Without S9 activation	μg/plate	With S9 activation	μg/plate
**TA97a**	4-nitro-o-phenylenediamine (NPD)	10.0	2-aminofluorene (2-AF)	4.0
**TA98**	4-nitro-o-phenylenediamine (NPD)	10.0	Benzo[*a*]pyrene (BP)	4.0
**TA100**	Sodium azide (SA)	0.4	2-aminofluorene (2-AF)	4.0
**TA102**	Mitomycin C (MMC)	0.5	2-aminoanthracene (2-AA)	4.0
**TA1535**	Sodium azide (SA)	0.4	2-aminoanthracene (2-AA)	4.0

### *In vitro* chromosomal aberration (CA) assay

To assess mutagenicity, the *in vitro* CA assay was measured according to procedures described in OECD Guideline 473 (1997) [20] and the US Food and Drug Administration’s Good Laboratory Practice (FDA GLP) guidelines (2002) [21] by counting and scoring the Chinese hamster ovary cells CHO-K1 with CA for *L*. *mali* APS1 treatment and the control in the presence or absence of S9.

To assess cytotoxicity, the *L*. *mali* APS1 was diluted with culture medium to final concentrations of 0.3125, 0.625, 1.25, 2.5, and 5.0 L mg/mL, and the cells were treated with or without the metabolic activation system (extracts were incubated with the S9-fraction for 3 h before treatment). Two mutants, 2-μM mitomycin C (Sigma-Aldrich, MO, USA) and 80-μM cyclophosphamide monohydrate (Sigma-Aldrich, MO, USA), were used as the positive control reagents in the absence and presence of S9 fractions, respectively. Without S9 metabolic activation, all groups were treated for a short-term (3 h) or continuous (20 h) period.

Finally, 100 metaphases were scored for each group. The number of aberrated cells, which was 5% greater than the positive control group, was determined as a positive result.

### *In vivo* micronucleus (MN) assay

The mammalian *in vivo* MN test was carried out according to the OECD Guideline 474 (1997) [22]. Twenty-five Six-week-old male ICR mice were housed in cages (5 per cage) with aspen chips (Nepco, U.S.A.) bedding subjected to 12 h light/dark cycle (06:00–18:00 h lighting period) under appropriate environmental conditions (22°C, 50 ± 20% relative humidity). Laboratory Autoclavable Rodent Diet 5010 (LabDiet, PMI Nutrition International, USA) and water were provided *ad libitum*. Following one week of acclimation, five mice were allocated randomly to each group and were given three different concentrations of *L*. *mali* APS1 (500, 1,000 or 2,000 mg/kg b.w.) by daily oral gavage. Distilled water and 1.0 mg/kg mitomycin C (Sigma-Aldrich, MO, USA) were used as the negative and positive controls, respectively. For MN assay, mice were anaesthetised by inhalation of isoflurane and peripheral blood samples were collected from orbital sinus at 48 and 72 h after dosing, the positive control group was only sampled at 48 h after dosing. Further analyzed for reticulocytes (RETs) cell counting, 10μL blood samples were smeared on a glass slide coated with 1% brilliant cresyl blue (BCB, Sigma-Aldrich, MO, USA) and analyzed with a Nikon ECLIPSE E600 optical microscope at 400× magnification. The RETs among total erythrocytes ratio was calculated by counting 1,000 erythrocytes per animal. To identify positive MN, 5 μL blood samples were smeared on acridine orange (Sigma-Aldrich, MO, USA)-coated slides for 4 h at 4°C, and further analyzed the ratio of MN (stained yellow-green) in 2,000 RETs (stained red) per animal under a florescence microscope (Leica, DM4000B, Wetzlar, Germany).

### Developmental toxicity study

*L*. *mali* APS1 was evaluated in a prenatal developmental toxicity study with sexually mature (8 weeks old) female SD rats according to OECD Guideline 414 (2001) [23]. Four animals of the same sex were housed in the cages with the same condition in MN test before mating, two females and one male animal per cage during mating. Following positive evidence of mating (day 0), the pregnant females were individually housed with soft nesting material. Test animals were provided continuous access to tap water and Laboratory Autoclavable Rodent Diet 5010. *L*. *mali* APS1 was administered orally to groups of pregnant female rats (20 per group) daily from days 6 to 15 of gestation at doses of 500, 1,000, and 1,670 mg/kg b.w./day, with a dosage volume of 10 mL/kg. The daily equivalent volume of distilled water was given to the animals in the control group. All animals were observed once daily for mortality and clinical observations. During pregnancy, the body weight of each rat was recorded prior to treatment on days 0, 6 to 15, 18, and 20. The quantity of food consumption (g/rat/day) was recorded on days 6, 9, 12, 15, 18, and 20.

On the day before parturition (day 20 of gestation), all female rats were sacrificed by CO_2_ asphyxiation to collect fetuses. After opening the uteri, all fetuses were examined for external abnormalities via gross visual observation. The uterine weight and numbers of all fetuses, viable or dead fetuses, total resorptions, corpora lutea, and implantation sites were examined. The fetuses were removed from the uterus, and the implantation site and the number of viable fetuses were recorded. The fetuses were weighed, sexed, and examined for external abnormalities. Half of the viable fetuses were fixed in Bouin’s fluid whereas the remaining half in Alizarin Red S (Sigma-Aldrich, MO, USA) to examine for visceral and skeletal malformations, respectively.

### Statistical analyses

Data were presented as mean ± SD. The statistical analysis was carried out using one-way analysis of variance (ANOVA) with Dunnett's multiple test. In the MN test, the statistical analysis was performed using one-way ANOVA followed by the Mann-Whitney test. Significant differences in MN frequency were calculated using the Kruskal-Wallis test between the negative control and treatment groups. Differences were considered significant at P<0.05 for all assays and were carried out using SigmaPlot 12 software (Systat software Inc., CA, USA).

## Results

### Ames test

The genotoxicity was conducted by bacterial reverse mutation assay with different doses of *L*. *mali* APS1 against five mutant *S*. *typhimurium* strains. An expected increase of revertant colonies was observed in all positive groups after induction by the mutants. Compared to the negative control, after exposure of bacterial strain to different concentrations of *L*. *mali* APS1, no significant (p>0.05) increase in the number of revertant colonies was observed in the presence or absence of S9 (Table 2). Therefore, the *L*. *mali* APS1 treatment groups were considered no mutagenic activity in the histidine auxotrophy of the *S*. *typhimurium* strains.

**Table 2:** Ames test results of *L*. *mali* APS1 powder using *Samonella typhimurium* strains TA97a, TA98, TA100, TA102, and TA1535.

*L*. *mali* APS1(mg/plate)	Number of revertant/plate				
	TA97a	TA98	TA100	TA102	TA1535
**With S9**					
5.0000	144.0 ± 7.0	32.7 ± 3.2	149.7 ± 9.2	369.3 ± 42.0	15.3 ± 0.6
2.5000	139.3 ± 4.0	31.3 ± 2.5	132.7 ± 11.4	426.0 ± 71.4	14.7 ± 2.9
1.2500	142.0 ± 4.0	24.7 ± 3.2	156.0 ± 15.6	437.3 ± 16.7	16.7 ± 3.1
0.6250	146.0 ± 11.3	27.0 ± 2.6	146.7 ± 10.4	444.0 ± 35.6	18.7 ± 1.5
0.3125	148.7 ± 13.9	26.7 ± 0.6	160.3 ± 11.8	449.3 ± 30.3	20.7 ± 2.1
Negative control^a^	151.7 ± 10.3	26.0 ± 0.0	151.7 ± 3.1	393.3 ± 18.9	21.7 ± 3.1
Positive control^b^	728.0 ± 25.5 *	1200.0 ± 0.088*	647.7 ± 41.6*	2226.7 ± 132.0*	372.7 ± 62.8*
**Without S9**					
5.0000	193.0 ± 21.4	35.3 ± 5.9	146.0 ± 5.2	413.3 ± 22.7	14.0 ± 1.7
2.5000	180.7 ± 14.8	31.7 ± 3.5	158.0 ± 4.4	488.0 ± 18.3	17.0 ± 1.7
1.2500	182.7 ± 13.6	28.7 ± 2.1	141.7 ± 7.6	500.0 ± 32.7	13.7 ± 1.2
0.6250	184.0 ± 11.1	30.7 ± 2.3	135.3 ± 3.1	530.7 ± 22.0	19.7 ± 1.5
0.3125	199.0 ± 10.5	33.7 ± 1.5	163.0 ± 11.3	488.0 ± 4.0	16.7 ± 3.5
Negative control^a^	187.0 ± 19.2	28.3 ± 2.1	157.3 ± 5.8	425.3 ± 57.5	21.0 ± 2.6
Positive controls^c^	815.3 ± 29.9*	322.7 ± 50.0*	354.7 ± 67.4*	930.7 ± 34.0*	211.7 ± 9.3*

Data were presented as the mean ± SD in three independent experiments.

*A significant difference compared to the negative control (p<0.05).

^a^ Sterile water was used as negative control.

^b^ Positive controls without S9 for TA97a, TA98: 4-nitro-o-phenylenediamine (NPD), 10.0 μg/plate; for TA100, TA1535: Sodium azide (SA), 0.4 μg/plate; and for TA102: Mitomycin C (MCC) 0.5 μg/plate.

^c^ Positive control with S9 for TA97a, TA100: 2-aminofluorene (2-AF), 4.0 μg/plate; TA98: Benzo[*a*]pyrene, 4.0 μg/plate; TA102, TA1535: 2-aminoanthracene, 4.0 μg/plate.

### Chromosomal aberration assay

The cytotoxicity data showed that CHO-K1 viability in short-term (3 h) treatment with *L*. *mali* APS1 was higher than 70.8 and 76.2% under the presence or absence of S9 activation, respectively. Moreover, the cell viability after continuous induction (20h) with *L*. *mali* APS1 without S9 activation was greater than 62.8%.

As shown in Table 3, chromosomal aberrated cell numbers in positive control groups treated with 2 μM of mitomycin C were higher than those of the negative control and *L*. *mali* APS1 treatment groups. Without the presence of the S9 activation, the frequency of aberrant chromosomes of CHO-K1 cells treated with 0.0315–5 mg/mL *L*. *mali* APS1 after induction for 3 h or 20 h were in the range of 0 to 0.5% and 0.5 to 3.5%, respectively. Under the same condition, the aberrant chromosomes for the positive control increased in 22 and 14%, respectively, as compared with the negative control. In the presence of S9 activation, the frequency of CA was 0.5 to 1.5% for induction of *L*. *mali* APS1, while the positive control was 15.1%. In all experimental conditions, the CA frequency was lower than 5% in comparison with control, suggesting that *L*. *mali* APS1 was safe in terms of genotoxicity at doses up to 5.0 mg/mL.

**Table 3:** Effect of *L*. *mali* APS1 on chromosomal aberration counts in 200 metaphase CHO-K1 cells.

*L*. *mali* APS1(mg/mL)	AC (%)^a^	Number of chromosome aberrations/200 cells													
		SG	SB	SD	TG	TB	TD	TR	QR	R	CR	DC	PP	PC	AF^b^
**-S9, 3h**															
5.0	0	0	0	0	0	0	0	0	0	0	0	0	0	0	0/200
2.5	0	0	0	0	0	0	0	0	0	0	0	0	0	0	0/200
1.25	0	0	0	0	0	0	0	0	0	0	0	0	0	0	0/200
0.625	0	0	0	0	0	0	0	0	0	0	0	0	0	0	0/200
0.3125	0.5	0	0	0	0	0	0	1	0	0	0	0	0	0	1/200
Negative Control^c^	0	0	0	0	0	0	0	0	0	0	0	0	0	0	0/200
Positive Control^d^	22	0	0	0	0	0	0	31	13	0	0	0	0	0	44/200
**-S9, 20h**															
5.0	3.5	0	0	0	0	0	0	0	1	0	0	1	5	0	7/200
2.5	1	0	0	0	0	0	0	0	0	0	0	0	2	0	2/200
1.25	0	0	0	0	0	0	0	0	0	0	0	0	0	0	0/200
0.625	0	0	0	0	0	0	0	0	0	0	0	0	0	0	0/200
0.3125	0.5	0	0	0	0	0	0	1	0	0	0	0	0	0	1/200
Negative Control^c^	0	0	0	0	0	0	0	0	0	0	0	0	0	0	0/200
Positive Control^d^	14	0	0	0	0	0	1	17	10	0	0	0	0	0	28/200
**+S9, 3h**															
5.0	1.5	0	0	0	0	0	0	1	0	0	0	0	2	0	3/200
2.5	0.5	0	0	0	0	0	0	0	0	0	0	0	1	0	1/200
1.25	0	0	0	0	0	0	0	0	0	0	0	0	0	0	0/200
0.625	0	0	0	0	0	0	0	0	0	0	0	0	0	0	0/200
0.3125	0.5	0	0	0	0	0	0	0	0	0	0	0	1	0	1/200
Negative Control^c^	0	0	0	0	0	0	0	0	0	0	0	0	0	0	0/200
Positive Control^e^	15.1	0	0	0	2	1	19	9	0	0	0	0	0	0	31/200

SG: chromosome gap, SB: chromosome break, SD: chromosome deletion, TG: chromatid gap, TB: chromatid break, TD: chromatid deletion, TR: triradial, QR: quadriradial, R: ring, CR: complex rearrangement, DC: dicentric, PP: polyploid, PC: pulverized cell. Chromosome gaps were recorded separately but not included in aberrant cells.

^a^ AC: The percentage of cells with chromosomal aberration in 200 metaphase cells (n/200).

^b^ AF: The number of cells with chromosomal aberration in 200 metaphase cells (n/200).

^c^ A culture medium with 10% fetal bovine serum.

^d^ 2 μM Mitomycin C was used without S9

^e^ 80 μM cyclophosphamide monohydrate was used with S9.

### Genotoxicity assay

During the three-day experimental period, no significant animal mortality, clinical symptoms, or abnormal body weight changes were observed. Treatment with mitomycin C as the positive control did not reduce the RETs/total erythrocytes in 48 (12.0 ± 1.6) or 72 h (9.0 ± 1.7) of observation (Table 4). Conversely, *L*. *mali* APS1 did not change the frequency of RETs in total erythrocytes at any dose tested on animals and showed no significant difference in the negative control group (p>0.05). The positive control significantly increased (p<0.05) in the frequency of micronucleated RETs (20.6 ± 1.7) when compared with the negative control and treatment groups (Table 4). Additionally, *L*. *mali* APS1 did not increase the MN% in RETs at any dose tested on all animals, indicating no significant difference as compared to the negative group (p>0.05).

**Table 4:** Changes of micronucleus counts in peripheral blood of ICR mice treated with *L*. *mali* APS1 powder.

*L*. *mali* APS1 dose(mg/mL)	Time (h)	RET/1000 RBC						MN/2000 RET					
		1^c^	2	3	4	5	Mean ± S.D.	1	2	3	4	5	Mean ± S.D.
Low (500 mg/kg b.w./day)	48	30	33	32	41	33	33.8 ± 4.2	1	1	3	1	2	1.6 ± 0.9
	72	27	33	34	28	34	31.2 ± 3.4	2	0	2	2	0	1.2 ± 1.1
Middle (1000mg/kg b.w./day)	48	30	32	34	33	32	32.2 ± 1.5	1	1	2	0	2	1.2 ± 0.8
	72	27	32	37	34	28	31.6 ± 4.2	1	0	2	2	1	1.2 ± 0.8
High (2000 mg/kg b.w./day)	48	31	30	31	33	33	31.6 ± 1.3	0	1	0	0	1	0.4 ± 0.5
	72	33	30	33	37	32	33.0 ± 2.5	0	1	3	0	1	1.0 ± 1.2
Negative control^a^	48	33	35	38	39	35	36.0 ± 2.4	1	0	1	1	0	0.6 ± 0.5
	72	32	36	31	38	32	33.8 ± 3.0	0	2	0	4	1	1.4 ± 1.7
Positive control^b^	48	10	12	13	11	14	12.0 ± 1.6	23	21	19	21	19	20.6± 1.7*
	72	6	9	10	10	10	9.0 ± 1.7	-	-	-	-	-	-

Data were presented as the mean ± SD (n = 5). RET: reticulocyte, RBC: total erythrocyte, MN: micronucleus.

*A significant difference compared to the negative control (p<0.05).

^a^ Sterile water was used as negative control.

^b^ 2 μM mitomycin C induction.

^c^ Serial number of test animal.

### Developmental toxicity study

No mortality and test substance-related clinical observation were noted in all dosage groups of animals studied. All test animals exhibited an increase in body weight during the study period. The changes in food consumption (g/rat/day) of maternal rats during the pregnancy period was not statistically significant as compared to the controls (p>0.05).

On the day before parturition (day 20 of gestation), no external abnormalities via gross visual observation were observed for any of the test groups (Table 5). Mean maternal uterine weight, implantation sites, total fetuses, viable fetuses, total resorptions, numbers of male fetuses, ratio of male to female fetuses (M:F) and ratio of pre- and post-implantation loss in all *L*. *mali* APS1 treated groups were similar to those in controls (p>0.05). The number of male fetuses was significantly (p<0.05) higher than that of the control group but showed no dose-related response. The M : F ratio showed no statistical significance. Moreover, no dead fetuses were observed in any of the test groups.

**Table 5:** Effect of *L*. *mali* APS1 on developmental toxicity study in maternal rats showing an overview of the effects on the main observed reproductive toxicity.

Items	Test groups			
	Control (0 mg/mL)	Low (500 mg/kg b.w./day)	Middle (1,000 mg/kg b.w./day)	High (1,670 mg/kg b.w./day)
No. of test animals	20	20	20	20
Maternal mortality (n/n)^a^	0/20	0/20	0/20	0/20
Clinical observation (n/n)^a^	0/20	0/20	0/20	0/20
Coarse lesion (n/n)^a^	0/20	0/20	0/20	0/20
0-20d body weight change (g)^b^	381.40 ± 28.20	374.50 ± 30.30	381.40 ± 31.90	380.20 ± 27.20
0-20d food consumption (g)^b^	555.90 ± 60.79	533.80 ± 42.30	555.85 ± 57.12	544.95 ± 40.90
Uterine weight (g)	79.20 ± 16.99	77.74 ± 15.04	81.08 ± 13.46	83.72 ± 9.85
No. of corpora lutea	18.10 ± 2.80	17.00 ± 3.80	17.30 ± 2.50	16.60 ± 2.90
No. of implantation sites	14.40 ± 03.10	14.30 ± 2.50	14.50 ± 2.50	4.50 ± 1.50
Pre-implantation loss (%)^c^	20.37 ± 15.98	14.12 ± 13.53	15.58 ± 13.42	10.88 ± 10.73
Post-implantation loss (%)^d^	3.11 ± 5.72	8.34 ± 10.08	4.64 ± 7.42	1.80 ± 3.20
No. of all fetuses	13.90 ± 2.90	13.20 ± 3.00	13.80 ± 2.40	14.30 ± 1.60
No. of viable fetuses	13.90 ± 2.90	13.20 ± 3.00	13.80 ± 2.40	14.30 ± 1.60
No. of total resorptions	0.50 ± 0.90	1.10 ± 1.00	0.70 ± 1.20	0.30 ± 0.40
No. of male fetuses/litter	8.20 ± 2.50	6.40 ± 2.00*	6.80 ± 2.10	8.10 ± 1.70
No. of female fetuses/litter	5.7 ± 2.4	6.90 ± 1.90	7.10 ± 2.10	6.20 ± 1.90
M:F^e^	2.11 ± 2.70	1.01 ± 0.49	1.09 ± 0.56	1.52 ± 0.77

Data were presented as the mean ± SD (n = 20).

*A significant difference compared to the negative control (p<0.05).

^a^ No. of abnormal animals/No. of total animals.

^b^ Observation period from the day of positive evidence of mating (day 0) to the day before parturition (day 20).

^c^ Pre-implantation loss % = (No. of corpora lutea–No. of implantation sites)/No. of corpora lutea x 100

^d^ Post-implantation loss % = (No. of implantation sites–No. of fetuses)/No. of implantation sites x 100

^e^ M: male fetus, F: female fetus.

The total number examined for external abnormalities were 277 (control), 264 (low-dosage), 276 (middle-dosage), and 285 (high-dosage) throughout the study period (Table 6). There were no adverse alterations of fetus development via gross visual observation, except for one pup with imperforate anus and one pup without a tail in the middle-dosage group and high-dosage group, respectively.

**Table 6:** Incidence of fetal abnormalities after administration of *L*. *mali* APS1 to maternal rats (F_0_).

Items	Test groups							
	Control (0 mg/mL)		Low (500 mg/kg b.w./day)		Middle (1,000 mg/kg b.w./day)		High (1,670 mg/kg b.w./day)	
Total fetuses	277		264		276		285	
Total No. examined for external abnormalities (n)	277		264		276		285	
Total No. examined for visceral abnormalities (n)	132		124		136		139	
Total No. examined for skeletal abnormalities (n)	145		140		140		146	
Fetal weight (g, mean ± SD)	3.80 ± 0.17		4.00 ± 0.21		3.84 ± 0.22		3.94 ± 0.20	
Fetal length (mm, mean ± SD)	36.96 ± 0.28		37.01 ± 0.51		37.62 ± 0.49		37.49 ± 0.43	
No. of abnormality (n)	0		0		1		1	
**Visceral abnormalities (%)**	**L**^**a**^	**F**^**a**^	**L**	**F**	**L**	**F**	**L**	**F**
Dilatation of ventricular atrium	0.0	0.0	0.0	0.0	0.0	0.0	0.0	0.0
Cervical thymic remnant	25.0	5.3	10.0	1.6	20.0	5.1	40.0	6.5
Dilatation of renal pelvis	25.0	3.8	25.0	4.0	20.0	2.9	35.0	8.6
**Skeletal abnormalities (%)**	**L**^**a**^	**F**^**a**^	**L**	**F**	**L**	**F**	**L**	**F**
14th rudimentary rib(s)	45.0	8.3	25.0	7.1	30.0	8.6	30.0	8.9
Bipartite ossification of sternebra	5.0	0.7	5.0	1.4	0.0	0.0	30.0	4.8

^a^ L: per litter incidence, F: per fetal incidence.

The numbers of fetuses (litters) available for visceral abnormality evaluation were 132 (20), 124 (20), 136 (20), and 139 (20) in the control, 500, 1,000, and 1,670 mg/kg/day groups, respectively. No significant differences (p>0.05) of visceral abnormalities were observed in the control group or any of the *L*. *mali* APS1-treated groups, but a total of two intergroup differences, including cervical thymic remnant and dilatation of renal pelvis were noted (Table 6). Because these abnormalities were not dose-related with positive correlation and were not statistically significantly different (p>0.05) as compared with the control group, the findings were not considered to be *L*. *mali* APS1-related.

The numbers of fetuses (litters) available for skeletal abnormality evaluation were 145 (20), 140 (20), 140 (20), and 146 (20) in the control, 500, 1,000, and 1,670 mg/kg b.w./day groups, respectively. Data presented in Table 6 demonstrated that no specific variation in skeletal malformations was noted in the control or *L*. *mali* APS1-treated groups. Only one skeletal developmental variation consisting of bipartite ossification of the sternebra was observed. The finding was not considered dose-related with positive correlation. The distribution appeared to be random.

## Discussion

Concerning the safety of *L*. *mali* APS1 for human consumption, the mutagenicity and genotoxic properties were first evaluated *in vitro*. We did not observe any treatment-related mutagenic activity in the histidine auxotrophy of the *S*. *typhimurium* strains up to 5.0 mg/plate. The *Salmonella* tester strains dock different mutations (hisD3052, hisG46, hisC3076, hisG428, hisD6610 and hisO1242) in the genes of the histidine operon with an rfa mutation for permeation of test chemicals. With the exception of TA102, all *Salmonella* testers have a deletion mutation of the uvrB gene to keep adducts generated with test chemicals as well as gal, chl, and bio genes [24, 25]. Other LAB strains, such as *L*. *paracasei* subsp. *paracasei* NTU 101 [26, 27], *L*. *plantarum* in TA98 and TA1535 [28], and a mix of probiotics containing *L*. *rhamnosus* LCR177, *B*. *adolescentis* BA286, and *P*. *acidilactici* PA318, also showed no mutagenic activity under analogous conditions based on the OECD Guidelines. No toxicity and mutagenicity in *L*. *mali* APS1 were observed in CHO-K1 cells, which have a short population doubling time and a stable karyotype of 20/21 chromosomes. Karyotypic stability insures the functionally monosonic state of the HGPRT gene [29]. In general, most LAB strains showed anti-mutagenic activity. A wide spectrum of dosages (from 0.3125 to 5 mg/mL) of live or heat-killed *Lactobacillus* spp., including *L*. *plantarum*, *L*. *paracasei*, and *L*. *rhamnosus*, have been reported not to induce a significant increase in the frequency of mammalian cells with CAs when compared with the positive control [27, 30, 31]. However, *L*. *acidophilus* strain showed low level of anti-mutagenic activity in one study [32]. Although one single test might not be sufficient to challenge the validity of commonly used bacterial reverse mutation test of other LAB strains, additional *in vivo* tests such as the chromosome aberration assay and micronucleus assay were needed to further verify the strain’s genotoxicity and clastogenicity.

*In vivo*, we followed the recommendations of the European Food Safety Committee [33] to evaluate the genotoxic response of a mammalian peripheral blood micronucleus. No genotoxicity was observed on *L*. *mali* APS1 at doses up to 2,000 mg/kg b.w./day, which is the upper limit dose in similar studies conducted [31, 34], with no clinical signs and body weight changes on any of the tested animals. The exposure of cells to genotoxic substances is known to damage chromosome fragments and could lead to the formation of micronuclei [35, 36]. The MN count gives an indirect evaluation of potential genotoxicity damage at the chromosomal level [36–38]. Our data was consistent with the findings from several equivalent *in vivo* genotoxic studies of probiotics, which have demonstrated no genotoxic response on experimental animals at different maximum doses, ranging from 5,000 (a multispecies probiotic mixture containing *L*. *rhamnosus* LCR177, *B*. *adolescentis* BA286, and *Pediococcus acidilactici* PA318) to 16,720 mg/kg b.w./day (*L*. *paracasei* subsp. *paracasei* NTU 101) [27, 30, 31].

We further evaluated the developmental toxicity of *L*. *mali* APS1 in rats with doses ranging from 500 to 1,670 mg/kg b.w./day. No *L*. *mali* APS1-related external, visceral, and skeletal malformations or developmental variations were observed for the fetuses in any of the treatment groups. Few of fetuses showed visceral variation (cervical thymic remnant and dilatation of renal pelvis) with a 0.18% incidence rate. However, the incidence of external abnormalities in the equivalent to each group did not correlate with the treatment dosage; hence there was no statistical difference between control and the treatment groups. A higher incidence of external abnormalities (0.41%) was reported by Yakabe et al. [39] but also without significance between the treatment group and the control group. Other reports described cervical remnant of the thymus [39, 40] and dilated renal pelvis [41] in middle or high-dose groups, but the incidence did no differ between the control or low-dose groups. Equally noteworthy is that skeletal variations in normal rats have been reported, i.e., cervical ribs, separation of ossified centers of the sternebra, accessory sternebra, shortening of rib [39–42]. Likewise, a mild skeletal developmental abnormality consisting of bipartite ossification of the sternebra was observed in the control and treatment groups in this study. This finding did not only occur in the high-dose group and was not considered dose-related with positive correlation; the distribution appeared to be random. Overall, there was no indication that *L*. *mali* APS1 increased the frequency of mouse developmental toxicity. Based on the treatment-related reproductive toxicity at any dosage level for *L*. *mali* APS1 administered once daily orally by gavage to pregnant rats for 10 days (days 6–15 of gestation), a maternal and developmental NOAEL (no-observed-adverse-effect-level) greater than 1,670 mg/kg/day was identified for both the parental and F1 populations.

## Conclusion

The results obtained in this study using the Ames test, CA, and *in vivo* MN test showed that *L*. *mali* APS1 did not exhibit mutagenic and genotoxic effects at any dose. In the prenatal developmental toxicity study, the data supported the NOAEL for maternal treatment-related reproductive toxicity and fetal development when the *L*. *mali* APS1 dosage was 1,670 mg/kg b.w./day. This study demonstrated that *L*. *mali* APS1 is safe based on the lack of treatment-related adverse effects at the highest dose level. However, in-depth understanding of its subchronic oral toxicity and a 90-day oral toxicity study are required to confirm the safety of *L*. *mali* APS1 for prolonged consumption.
